# Variation of Functional Neurological Symptoms and Emotion Regulation with Time

**DOI:** 10.3389/fpsyt.2018.00035

**Published:** 2018-02-13

**Authors:** Johanna Kienle, Brigitte Rockstroh, Johanna Fiess, Roger Schmidt, Tzvetan Popov, Astrid Steffen-Klatt

**Affiliations:** ^1^Department of Psychology, University of Konstanz, Konstanz, Germany; ^2^Kliniken Schmieder, Konstanz, Germany

**Keywords:** functional neurological symptom disorder, somatoform dissociation, conversion disorder, emotion regulation, alexithymia, electroencephalography

## Abstract

**Introduction:**

The present study addressed the variation of emotion regulation in the context of functional neurological symptom disorder (FNSD) by examining changes of functional neurological symptoms (FNS), general psychological strain, alexithymia, emotion regulation strategies, and cortical correlates of emotion regulation in the context of a standard inpatient treatment program.

**Methods and materials:**

Self-report data on FNS, general psychological strain, alexithymia, emotion regulation strategies, and cortical correlates of an experimentally induced emotion regulation task (participants either passively watched unpleasant and neutral pictures or regulated their emotional response to unpleasant pictures using pre-trained reappraisal, while an electroencephalogram was recorded) were compared between 19 patients with FNSD and 19 healthy comparison participants (HC) before and after a 4-week standard treatment protocol that included a combination of (individual and group) psychotherapies and functional treatments (such as physiotherapy) or a 4-week interval in HC, respectively.

**Results:**

General psychological strain did not decrease significantly in FNSD patients. Changes in emotion regulation in FNSD patients were constrained to an increase in self-reported use of cognitive reappraisal strategies. Subjective symptom intensity in FNSD patients varied with alexithymia pretreatment, but did not decrease significantly. Cortical activity in the time and frequency-domain distinguished passive watching of neutral and unpleasant pictures and regulating emotional responses upon unpleasant pictures from passively watching them without difference between groups and/or time.

**Discussion:**

Over the investigated time interval, augmented habitual cognitive emotion regulation suggests an alleviation of emotion processing deficits, but no significant symptom decrease. More controlled and prolonged treatment studies would be needed to determine whether and how a specific contribution of treatment-related changes of emotion regulation and FNS might be inferred.

## Introduction

Functional neurological symptoms (FNS), that is, impaired voluntary motor or sensory function without verified neurological or medical basis (1) cause pervasive suffer and often long-term treatment (2, 3). While the traditional concept of these symptoms as conversion of emotional conflicts and psychological strain into physical expression (4) motivated the assignment of FNS to dissociative disorders [ICD-10 (5)], DSM-5 (1) acknowledges the specific pattern of symptoms and factors contributing to their generation with the distinct category “functional neurological symptom disorder” (FNSD). Hypotheses on symptom generation still consider dissociation and conversion within the concept of aberrant emotion processing consequent upon emotionally upsetting or traumatic experiences and/or emotional conflicts (6). Further clarification of emotion processing in FNSD should inform concepts and therapeutic procedures.

Framing FNSD as dissociative and conversion disorder linked emotion processing primarily to impairments in the perception and verbal expression of one’s own feelings, as conceptualized by alexithymia (7). Framing emotion processing in the context of stress and coping extended emotion regulation to cognitive and behavioral strategies that aimed at (explicit or implicit) control of unpleasant feelings in response to external events; major strategies are described and measured as cognitive reappraisal of the upsetting event or suppression of rising unpleasant sensation (8, 9). Both alexithymia and altered emotion regulation have been verified for patients with FNS: elevated levels of alexithymia (10–12) were explained as a transformation of emotional expressions into bodily symptoms or as a misperception of autonomic concomitants of emotion (i.e., increased heartbeat during the experience of fear) as signs of physical illness (10). Altered emotion regulation was verified as stronger tendency for emotion suppression and less cognitive reappraisal in FNSD patients than in healthy controls (13).

In addition to self-reported alexithymia and emotion regulation strategies brain dynamics measured in experimental emotion regulation tasks aimed at substantiating the role of emotion regulation in FNSD. Hemodynamic and electromagnetic imaging studies confirmed altered processing of emotional stimuli and emotion regulation in FNSD compared to controls in those frontocortical and parietal cortices that have been related to emotion regulation (14, 15). In addition to frontal abnormality, augmented activity in movement-related cortical areas was reported for patients with conversion disorder compared to controls (16–19). Similarly, Fiess et al. (18) reported less frontocortical but augmented sensorimotor electromagnetic activity in FNSD compared to controls in an emotion regulation task, in which participants implemented cognitive reappraisal strategies when watching unpleasant stimuli.

The contribution of emotion processing to FNS generation may be further probed in the context of treatment. Many treatment programs target emotion regulation in conflict situations, considering symptoms as manifestation of dysfunctional emotion regulation or emotional conflict resolution by conversion into physical expression [brief psychodynamic interpersonal therapy (20, 21); enriching cognitive behavior therapy with emotion regulation (22)]. Thus, variation with treatment may test hypotheses on the role of emotion regulation in FNSD. Moreover, while so far an increasing research body on risk factors and/or comorbid symptoms and other characteristics of FNS(D) can be observed (see above), little is known of their interaction and/or variation over time—or even over treatment.

To this end, the present study combined self-reported alexithymia and emotion regulation strategies and cortical indices of experimentally induced emotion regulation in the time-domain (*via* event-related potentials; ERP) and in the frequency-domain between FNSD patients and healthy controls to gain further insight in altered emotion regulation in FNSD. In addition, the indices of emotion processing were examined before and after a standard clinical treatment program for FNSD that addressed emotion regulation, emotional conflict awareness, and analysis and resolution of learned stimulus-symptom contingencies as potential contribution to symptom generation.

Specific hypotheses were (1) FNSD patients express more alexithymia, psychological strain, and use less cognitive reappraisal and more suppression when downregulating emotions than healthy comparison participants (HC) prior to treatment. (2) FNSD patients recruit less frontal and more sensorimotor EEG activity than HC while applying cognitive reappraisal in an experimental emotion regulation task prior to treatment. (3) In FNSD, subjective and cortical indices of emotion regulation change across time in parallel to a change in symptom severity and overall psychological strain.

## Materials and Methods

### Participants

Twenty-six patients with FNSD were recruited at the neurological rehabilitation centers Kliniken Schmieder in Konstanz and Gailingen. Patients were diagnosed with ICD-10 diagnoses of a dissociative disorder (ICD-10 code F44.4: dissociative movement disorder, F44.6: dissociative sensibility disorder, F44.7: mixed dissociative disorder), which corresponds to the DSM-5 diagnosis FNSD. Individual diagnoses were given by at least two psychiatrists and neurologists according to ICD-10 guidelines (5). Patients with a history of central nervous lesion or disorder were excluded, and at least one core negative FNS (e.g., paresis or hypesthesia) was required for inclusion in the study. *N* = 5 of the 26 FNSD patients did not complete the study (*n* = 3 due to early discharge, *n* = 2 because of a change in diagnosis). In addition, data of two patients were excluded from analyses because of artifact-contaminated EEG data. Table 1 summarizes demographic and clinical data of the remaining sample of *n* = 19 FNSD patients (*n* = 11 with diagnosis F44.7, *n* = 7 with diagnosis F44.4, *n* = 1 with diagnosis F44.6). Seven FNSD patients suffered from motor weakness or sensory disturbances of left-sided, *n* = 5 of right-sided limbs, in *n* = 7 both sides were affected. The average duration of inpatient admission was 4 weeks (33 ± 5.8 range 27–45 days). At the time of data assessment, about half of the patients in the present sample (10/19) received analgesic medication (of different active substance groups, only two received the identical drug). Three patients were unmedicated, 7 out of 19 received medication against hypertension and/or thyroid hypofunction. Only 3 out of 19 patients received an antidepressant. Following neurological and neuropsychiatric assessment, patients accomplished a multidisciplinary treatment program to reinstall a functional interaction of body perception and emotion processing [following standards as postulated, e.g., by Dallochio et al. (23) and Carson et al. (24)]. This program included psychoeducation (1 h per week), functional therapeutic (i.e., daily physiotherapy, general movement therapy, and depending on individual needs ergotherapy, logotherapy, or neuropsychological training—on average 9.25 h per week) and different psychotherapeutic interventions (on average 7.5 h per week, i.e., once to twice a week individual and four times a week group cognitive behavioral therapy, moreover once or twice a week art and/or bodypsychotherapy). Psychotherapy comprised behavioral therapy with elements of schema-, psychodynamic, and systemic therapies, and addressed emotional regulation within these frameworks.

**Table 1:** Sociodemographic information of study samples.

	FNSD patients	HC	FNSD patients vs. HC
*N*	19	19	38
Gender (f/m)	13/6	12/7	Chi^2^ = 0.12, *p* = 0.73
Age (M ± SD)	42.7 ± 14.2	50 ± 17.7	*t*(36) = 1,38, *p* = 0.17
Years schooling (M ± SD)	12.7 ± 4.5	14.3 ± 1.8	*t*(36) = 1,34, *p* = 0.18

*FNSD, functional neurological symptom disorder; HC, healthy comparison participants; f, female; m, male*.

Twenty-four HC were recruited in the local community by flyer and oral advertisement. Exclusion criteria were a central nervous lesion or disorder, and any psychological disorder as screened with the MINI international neuropsychiatric interview (25). After excluding *n* = 2 HC, who did not complete the study, and *n* = 2 with artifact-contaminated EEG, data of *n* = 19 HC were available for analyses. Groups did not differ in gender, age distribution, and years of education (see Table 1). All participants had normal or corrected to normal vision. Two healthy comparison participants were left-handed, patients diagnosed with FNSD were all right-handed.

### Study Design

The study design was approved by the Ethics committee of the University of Konstanz and by the IRB of the Kliniken Schmieder. All participants provided written informed consent prior to assessment onset in accordance with the Declaration of Helsinki. The study design addressed the two main hypotheses by comparison of dependent measures (FNS severity, self-rating, and electrocortical emotion regulation indices) between groups (FNSD patients and HC) and across time (before and after the inpatient treatment period in FNSD patients and a respective time interval in HC).

Dependent measures were assessed on three separate days: In a first session (for FNSD within 1 week after admission) participants were introduced into the study design and filled in questionnaires on demographic information, FNS severity and general psychological strain, alexithymia and habitual emotion regulation strategies. Two subsequent assessments involving the experimental emotion regulation task, during which the EEG was monitored, were scheduled before and after completion of the treatment program (patients) or after approximately 4 weeks (HC). The mean time interval between the two laboratory sessions was 23 ± 8.3 days (range 14–45 days) for FNSD patients and 33 ± 5.8 days (range 27–45 days) for HC. The respective time interval was significantly different between groups [*T*(36) = 4.5, *p* < 0.001].

### Data Acquisition and Analyses

#### Functional Neurological Symptoms

Functional neurological symptoms were assessed with the Somatoform Dissociation Questionnaire [SDQ-20 (26), German version by Mueller-Pfeiffer et al. (27)]. The SDQ-20 is a 20-item self-report instrument, which assesses the frequency of somatoform dissociation experienced in the preceding 12 months. Good internal consistency (Cronbach’s α = 0.92) and test–retest reliability (rtt = 0.89) are reported (27). In consideration of the 4-week treatment-interval assessment, a 1-week evaluation period was adopted for the present study, since changes were expected to be most pronounced in this time range. In addition, subjectively experienced symptom intensity was rated on an 11-point Likert scale with a range from 0 “no symptoms” to 10 “maximum intensity” pre and post each EEG session.

#### Psychological Strain

Psychological strain was evaluated with the Symptom-Checklist 90R [SCL 90R (28)]. The SCL90R includes 90 items that are rated on a five-point Likert scale and combined to nine subscales: somatization, obsessive-compulsiveness, interpersonal sensitivity, depression, anxiety, anger-hostility, phobic anxiety, paranoid ideation, and psychoticism. The mean score of items per subscale represents the respective dimension, while the general psychological strain load is represented by the mean score of all 90 items (global severity index, GSI). Good reliability is approved for subscales [between *r* = 0.75 and *r* = 0.87 (29)] and global indices [rtt = 0.68 to rtt = 0.80 (28)].

#### Emotion Regulation

Emotion regulation covered alexithymia, assessed with the Toronto Alexithymia Scale [TAS-26 (30), German version (31)], and the Emotion Regulation Questionnaire [ERQ (8), German version (32)]. The TAS-26 includes 26 self-report items that measure alexithymia on three dimensions: “difficulty identifying feelings,” “difficulty describing feelings,” and “externally oriented thinking.” Internal consistency (α = 0.84) and convergent validity are evaluated as good (31). The ERQ includes six items addressing cognitive reappraisal and four items addressing emotion suppression, each rated on a 7-point Likert scale, with good convergent validity and internal consistencies (32).

Differences between groups and assessments (pre-/posttreatment) in FNS- and emotion regulation indices were statistically evaluated by dependent-sample *t*-tests. Since the assumption of homogeneity of variance was not met, between-group differences were evaluated with non-parametric Mann–Whitney *U* tests. In FNSD patients, the relationships between changes in symptom severity and changes in emotion processing across the treatment period (post-pre difference scores of each scale) were probed using bivariate Pearson correlation analysis. To reduce the likelihood of a type I error, we applied a Bonferroni–Holm correction for multiple tests (33) and report only the corrected *p*-values.

#### Cortical Correlates of Emotion Regulation

Cortical correlates of emotion regulation were measured in an experimental emotion regulation task adopted from Fiess et al. (18) and adjusted to EEG monitoring: 70 high-arousing unpleasant and 70 low-arousing neutral pictures from the International Affective Picture System (34) were presented on a screen about 90 cm from the participant’s eyes. The 2-s picture presentation was preceded by a 2-s cue presentation (the capital letters A or R), indicating that participants should passively watch (A for the German word “Anschauen”) the respective picture or downregulate their emotional response to the picture using cognitive reappraisal (R for the German word “Regulieren”). Across the total 210 trials, the three conditions (watch neutral pictures, watch unpleasant pictures, and regulate emotions upon unpleasant pictures) were arranged in pseudorandom order (70 trials per condition). Intertrial intervals were jittered between 2 and 2.5 s. Following standard procedures [e.g., Moser et al. (35)] cognitive reappraisal strategies were practiced prior to the experiment with individually selected examples like “it’s a scene from a movie” or “help is coming.” As manipulation check participants were asked after the experiment, which strategy they had implemented and whether this had been successful.

#### EEG Data Acquisition and Analysis

##### Data Recording and Preprocessing

The EEG was recorded from 128 electrodes with active shielding placed on a Waveguard cap with equidistant hexagonal layout (ANT Neuro) using a direct-coupled amplifier (ANT). Signals were sampled with 2,048 Hz. A 40-Hz low pass 0-phase filter was applied offline. Impedances of all electrodes were kept below 20 kΩ. Data were preprocessed using FieldTrip, a matlab-based open-source signal processing toolbox (36). Epochs of 9 s length were extracted from the continuous recording (5 s pre-stimulus), time-locked to picture onset and corrected for cardiac, blink and eye-movement artifacts *via* independent component analysis (37).

##### ERP Analysis

A cluster-based, dependent-sample *F*-test with Monte-Carlo randomization (38) was calculated for both groups in a time interval of 0.4–1 s after picture onset to identify sensor clusters indicating significant condition differences (NW: passively watch neutral pictures, UW: passively watch unpleasant pictures, UR: regulate emotion upon unpleasant pictures). Selection of time of interest (TOI) followed evidence on the electroencephalographic late positive potential (LPP) 400 and 700 ms after stimulus onset over posterior regions that distinguishe implementation of cognitive reappraisal strategies upon unpleasant stimuli and passive watching of unpleasant stimuli (35, 39–41). Regions of interest (ROI) were identified on the basis of this cluster procedure. Averaged power across those ROI and TOI was analysed in two separate repeated-measures ANOVAs with the within subject factors condition and time (pre/posttreatment) and the between subject factor group.

##### Spectral Analysis

Time-frequency representations of the measured signal were obtained using Hanning-tapered sliding window with a fixed window length of 0.5 s, resulting in a 2 Hz frequency resolution. Stimulus evoked activity was expressed as change of power (in percent) relative to a cue-preceding baseline (−3 to −2.25 s) and averaged separately for group (HC, FNSD patients), conditions (NW, UW, UR), and time point (pre-/posttreatment or first–second assessment, respectively). Group differences in the modulation of oscillatory activity in a time-window from 0.3 to 2 s after picture onset in the frequency band of 8–12 Hz were evaluated using cluster-based dependent-sample *t*-tests with Monte-Carlo randomization^1^ (*N* = 1000; allowing the control of type 1 error rate in the context of multiple comparisons) with a 5% significance threshold for activity differences between sensor clusters (38). Time-window and frequency band were selected to be comparable to Fiess et al. (18) and Popov et al. (42). *Posthoc* planned comparisons confirmed the “emotion effect” as UW minus NW contrast, and the regulation effect as UR minus UW contrast. Group and time differences between were evaluated using independent-sample *t*-test statistics.

##### Source Analysis

Sources of activity that generated the effects at sensor level were determined by dynamical imaging of coherent sources beamformer on time-frequency windows that were defined based on the results at sensor level [DICS (43)]. Cross-spectral density matrices for a time period of 0.3–2 s after stimulus onset were calculated separately for conditions and groups using a multitaper method. The center frequency was set to 10 ± 2 Hz. A standard Montreal Neurological Institute-based (http://www.bic.mni.mcgill.ca/brianweb) boundary element method model (44) was used to create a realistic volume conduction model of the head. Electrodes were aligned using an ANT-specific template layout of the electrodes, based on averaged digitized electrode positions of 20 volunteers not included in the present report. Within-subjects, differences in source power for the “emotion effect” (NW − UW) and the “regulation effect” (UR − UW), were evaluated for the 0.3- to 2-s interval as defined above using dependent-sample *t*-tests, and group differences for each effect was examined by independent-sample *t*-tests. All analyses were two-sided with an alpha-level set to 0.05.

Relationships between changes in symptom severity scores, emotion regulation scores, and cortical emotion regulation indices across time/treatment were probed with bivariate Pearson correlation analysis.

## Results

### Pretreatment Group Comparison

Functional neurological symptom disorder patients reported higher FNS severity (SDQ-20: *U* = 48.5, *p* < 0.00) and higher psychological strain (SCL-90R: *U* = 22, *p* < 0.00) than HC. Emotion regulation in FNSD patients differed from HC with respect to more alexithymia than HC (TAS-26: *U* = 41, *p* < 0.00), less tendencies to use cognitive reappraisal (ERQ-R: *U* = 87.5, *p* < 0.006) but similar tendencies for emotion suppression (ERQ-S: *U* = 167.5, *p* = 0.71).

In the entire sample (FNSD patients and HC), symptom and emotion regulation measures were related, in that TAS-26 scores varied with SDQ-20 (*r* = 0.57, *p* < 0.001), SCL-90R GSI (*r* = 0.74, *p* < 0.001), and subjective level of symptom intensity in FNSD patients (*r* = 0.64, *p* < 0.001). Group-specific relationships were confirmed for TAS-26–SCL-90R GSI (*r* = 0.65, *p* = 0.003) in FNSD patients.

### Changes in the Context of Time/Treatment

Bonferroni–Holm adjusted effects confirmed significant changes over time/treatment only for increased use of cognitive reappraisal strategies in FNSD patients [ERQ-R, *t*(16) = −3.9, *p* = 0.001, here, the significant alpha-level after Bonferroni–Holm correction should be *p* < 0.0035], which did not differ from that in HC at the second assessment (*U* = 129, *p* = 0.3).

A slight decrease in symptom severity [SDQ-20, *t*(16) = 0.135, *p* = 0.9, following Bonferroni–Holm correction *p* < 0.05 would have been needed to reach significance], and psychological strain [SCL-90R from pre M ± SD.66 ± 0.48 to posttreatment M ± SD = 0.87 ± 0.5; *t*(16) = 0.14, *p* = 0.018, Bonferroni–Holm corrected *p* < 0.004 would have been needed] in FNSD patients did not reach significance. Hence, both symptom severity and psychological strain remained higher in FNSD than in HC (SDQ-20: *U* = 45.5, *p* < 0.001 SCL-90R: *U* = 46.5, *p* < 0.001) at the second assessment.

In FNSD patients, no significant changes can be reported for subjective symptom report [*t*(16) = 2.1, *p* = 0.05, Bonferroni–Holm corrected *p* < 0.007 would have been needed]. Similarly, alexithymia [*t*(16) = 1.37, *p* = 0.19, adjusted *p*, 0.01] and emotion suppression [*t*(16) = −0.45, *p* = 0.66, adjusted alpha-level: *p* = 0.02] did not vary with treatment and changes in symptom severity were not related with changes in emotion processing in FSND patients. For an overview on all reported Bonferroni–Holm adjusted alpha-levels please see Table S3 in Supplementary Material in Data Sheet S1 in Supplementary Material.

### Cortical Indices of Emotion Regulation

**Figure 1:**
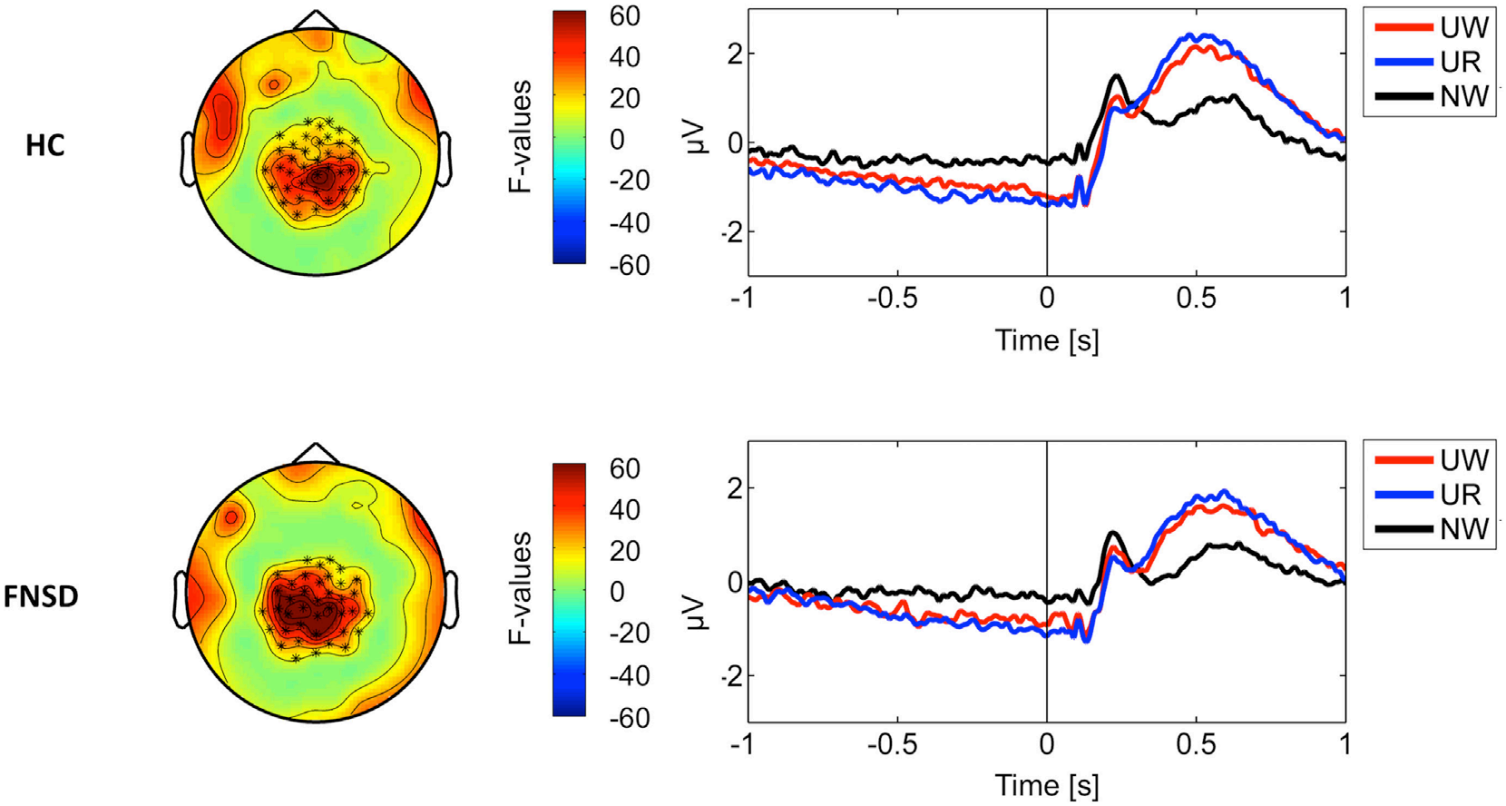
On the left part, sensor clusters indicating significant condition differences (marked by asterics). On the right part, corresponding time-courses of power of the measured signal averaged seperately over conditions (UW—passively watch unpleasant pictures, UR—regulate emotions upon unpleasant pictures, NW—passively watch neutral pictures) and groups [HC—healthy comparison subjects and functional neurological symptom disorder (FNSD)—patients with FNSD].

Figure 1 illustrates the identified ROI and corresponding time-course of the ERP during the stimulus–task interval separately for groups and conditions (passive watch, regulate). An “emotion effect” [*F*(1,36) = 107,16, *p* < 0.001] was indicated by the larger LPP in response to unpleasant compared to neutral stimuli in both groups. Similarly, a “regulation effect” was present in both groups [*F*(1,36) = 8,62, *p* < 0.01] with larger LPP during the regulation of unpleasant stimuli compared to the passive watching.

**Figure 2:**
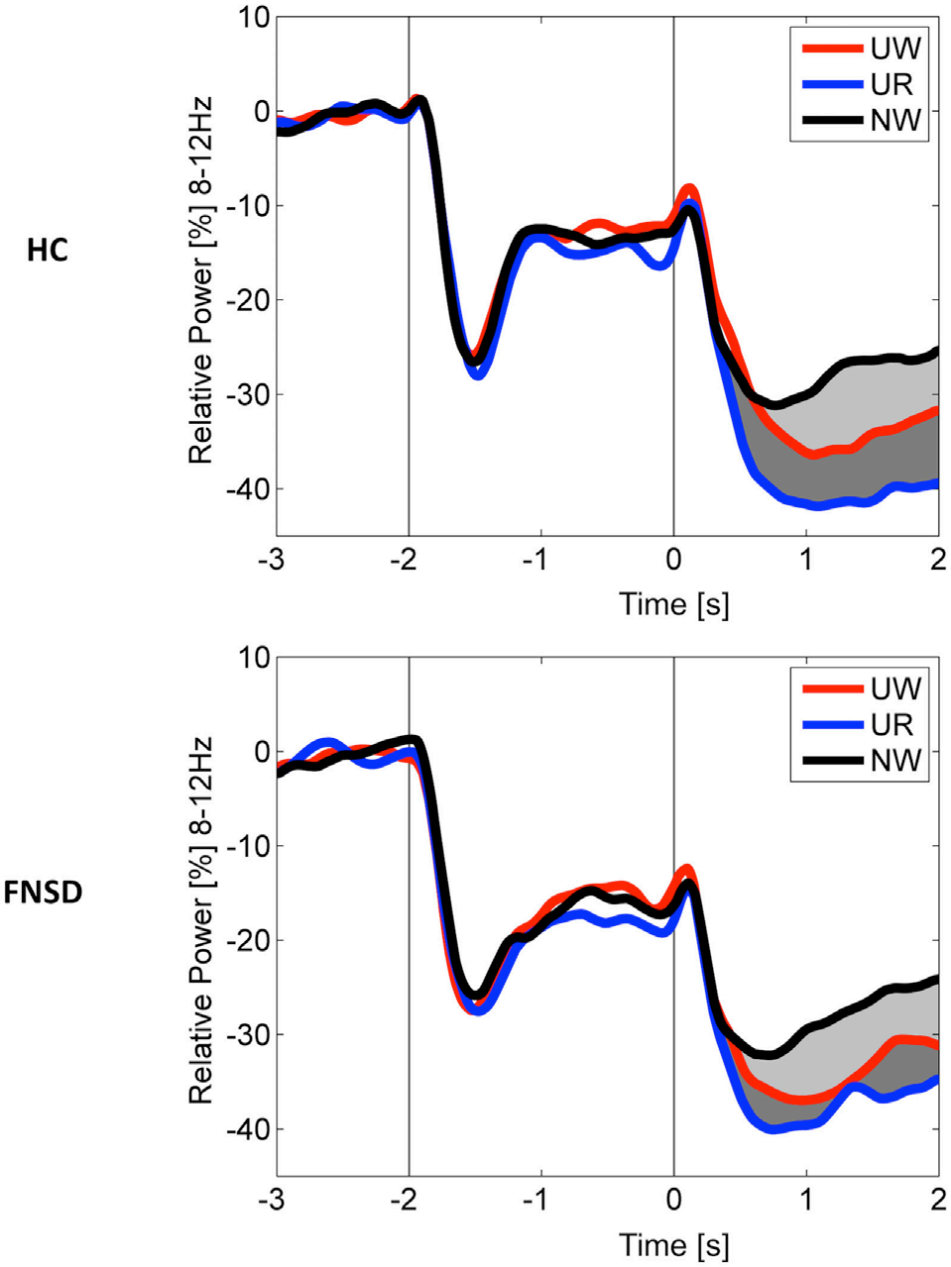
Grand average time-course of power in the 8–12 Hz band expressed as change (in percent) from pre-stimulus baseline (−3 to −2.25 s) for healthy comparison subjects (HC) and patients with functional neurological symptom disorder (FNSD). Time-courses of power changes during the picture interval (0–2 s) are averaged per group and condition (UW—passively watch unpleasant pictures, UR—regulate emotions upon unpleasant pictures, NW—passively watch neutral pictures). Light-gray shaded areas mark the emotion effect, dark-gray shaded areas mark the regulation effect.

Frequency-domain analyses (see methods) indicated event-related decrease in the 8–12 Hz alpha power in the 0.3- to 2-s time-window after stimulus onset relative to baseline. Figure 2 illustrates the time-course of event-related alpha power decrease across a 3-s baseline and 2-s stimulus interval separately for groups and conditions: In the pretreatment assessment, greater alpha power decrease during UW (watch unpleasant stimuli) than NW (watch neutral) trials characterized the “emotion effect” on sensor and source level in both groups (HC: *p* < 0.001, FNSD patients: *p* < 0.01). In the same time interval, the “regulation effect” was evident in both groups in marked alpha power decrease during UR compared to UW condition on sensor level (HC: *p* < 0.01; FNSD patients: *p* < 0.01). Neither “emotion effect” nor “regulation effect” changed over time (*p* = 0.34), indicating response stability in HC, and no impact of the treatment period on cortical correlates of emotion regulation in patients. However, within the FNSD sample, changes of the “regulation effect” across time varied with changes in the subjectively rated symptom severity (*r* = 0.52, *p* = 0.02) in that larger (post-minus pre treatment) changes in power between the conditions “watch unpleasant” and “regulate unpleasant” varied with larger changes in experienced symptom severity.

## Discussion

The present study evaluated indices of emotion regulation in relation to FNS by comparing these indices between FNSD patients and healthy controls and their variation with time and/or treatment. It was hypothesized (1) that groups differed in alexithymia, habitual emotion regulation style, and cortical responses during an experimental emotion regulation task before the treatment period and (2) that in FNSD patients changes in symptom severity and psychological strain after the treatment period varied with changes in emotion regulation indices. In support of the first expectation and in line with previous reports (10–12), FNSD patients exhibited more alexithymia and used less cognitive reappraisal strategies on everyday emotion regulation than controls, Relationships between emotion regulation indices and symptom expression were confirmed for the entire sample, but only for alexithymia and general psychological strain for FNSD patients. In addition, cortical correlates of “emotion” and “regulation” effects were replicated (18), whereas frontal and more sensorimotor cortex involvement in emotion regulation in FNSD patients (16, 18) was not replicated. The present small sample size and interindividual variability may have resulted in electrocortical responses overlapping between groups, preventing group differences.

Event-related potential analyses confirmed “emotion” and “regulation effects” in the expected cortical regions. In contrast to previous reports of smaller LPP during regulation than during passive watching (45, 46) the LPP was enhanced during the regulation of the emotional response upon unpleasant pictures. Similar evidence of increased LPP under regulation instructions was recently reported by Ellis et al. (47), suggesting that this increase may reflect allocation of attention upon the instruction to actively regulate upcoming emotions.

The expected changes over a period that included a standard treatment program for FNSD patients were not confirmed. Robust changes were only confirmed for an increased tendency to use cognitive reappraisal as emotion regulation strategy. The tendency to use suppressive strategies of emotion regulation did not change over time and did not differ between groups. These results are in line with previous evidence of an association between reappraisal but not suppression with physic and psychic health (48, 49). Moreover, results on cortical emotion regulation indices indicated their stability, but did not vary with treatment in FNSD patients.

Different factors may have contributed to the lack of significant differences between groups and over time: in addition to larger sample sizes for adequate statistical power, the short 4-week treatment program (i.e., following the standard health insurance coverage in Germany) may be insufficient to prompt substantial and sustained changes. The present small sample size constrained the comparison of FNSD patients who benefited from treatment or did not change. Treatment effects on emotion regulation strategies and their cortical correlates may emerge only with longer treatment periods or may develop over time even after the end of treatment, as shown by (50). Finally, more specific modules and targeted treatment may be a prerequisite for treatment-induced changes in emotion regulation on the different levels. So far, whether the self-reported increase of cognitive reappraisal to downregulate negative emotion reflects a substantial change in emotion regulation strategies or a trained reproduction of what was learnt in psychotherapy on how to (theoretically) regulate emotion without behavioral effects, remains unclear. Emotion regulation was just one target in the standard treatment program of the involved neurological rehabilitation center, and thus, the impact of intense treatment procedures focusing on emotion regulation remains to be evaluated. In consideration of these factors, the observed tendencies encourage hypotheses to be verified in future studies with longer, targeted treatment and follow-up assessment.

To conclude: probing the meaning of emotion regulation, measured on subjective and cortical level, in FNSD in the context of time/treatment offers first clues that need to be substantiated in powered, targeted studies: changes in the subjectively experienced symptom severity and psychological strain may vary with a tendency to adjust everyday emotion regulation toward accentuated use of cognitive strategies, and to reduce alexithymia. If substantiated, this should be considered in designing the concept of FNS and their generation, as well as for the adjustment of remediation strategies.

## Ethics Statement

The study design was approved by the Ethics committee of the University of Konstanz and by the IRB of the Kliniken Schmieder. All participants provided written informed consent prior to assessment onset in accordance with the Declaration of Helsinki.

## Author Contributions

AS-K and BR designed the project and, together with JK, JF, and RS, the study protocol. JK and JF collected the data under the supervision of AS-K and RS. JK, TP, and JF analyzed the data. JK, AS-K, and BR drafted the manuscript, and all authors contributed to the final version.

## Conflict of Interest Statement

The authors declare that the research was conducted in the absence of any commercial or financial relationships that could be construed as a potential conflict of interest.

## Footnotes

^1^This procedure begins with a t-test between every combination of time point and channel in the actual data and between the two groups. Adjacent time-channel-combinations that showed significant group differences in the t-tests were clustered by summing the t-values. A 1000-fold repetition of this procedure after randomly assigning group associations of each subject can be described as “Monte Carlo permutation,” resulting in a distribution with parallel results that is used for comparison with the original data: For each iteration, the t-value sum from the cluster with the highest t-value sum is added to a distribution reflecting the null hypothesis of no group difference. Finally, this hypothesis is evaluated by comparing the t-value sums of the actual data and the calculated distribution.
