# Supplementary material for: Variation of Functional Neurological Symptoms and Emotion Regulation with Time
Source: Front Psychiatry. 2018 Feb 13;9:35. doi: 10.3389/fpsyt.2018.00035 (PMC5816796; doi:10.3389/fpsyt.2018.00035)
Supplement: Supplementary file 1 [file table_2.docx]

| **Supplementary Table 2:** Mean and standard-deviation of alexithymia and emotion regulation (i.e. cognitive reappraisal and emotion suppression), pre-treatment. | | |
| --- | --- | --- |
|  | FNSD patients | HC |
| *N* | 19 | 19 |
| Alexithymia (TAS-26) | 2.89 ± .53 | 2.15 ± .35 |
| Cognitive reappraisal (ERQ_R) | 3.45 ± 1.54 | 4.75 ± 1.35 |
| Emotion suppression (ERQ_S) | 3.57 ± 1.93 | 3.26 ± .88 |
