# Supplementary material for: Variation of Functional Neurological Symptoms and Emotion Regulation with Time
Source: Front Psychiatry. 2018 Feb 13;9:35. doi: 10.3389/fpsyt.2018.00035 (PMC5816796; doi:10.3389/fpsyt.2018.00035)
Supplement: Supplementary file 2 [file table_3.docx]

| **Supplementary Table 3:** Bonferroni-Holm corrected alpha level and original p-values for the reported measurements | | |
| --- | --- | --- |
| Dimension (scale) | Bonferroni-Holm corrected alpha level | p-value |
| Symptom^[[1]](#footnote-1)^ severity (SDQ-20) | 0.05 | 0.89 |
| Subjective symptom report (self report, Likert scale) | 0.007 | 0.053 |
| Psychological strain (SCL-90R_GSI) | 0.004 | 0.018 |
| Alexithymia (TAS-26) | 0.01 | 0.19 |
| Cognitive reappraisal (ERQ_R) | 0.0035 | 0.001 |
| Emotion suppression (ERQ_S) | 0.02 | 0.65 |

1. Sypmptom = functional neurological symptom (FNS). [↑](#footnote-ref-1)
